# The Putative Role of mTOR Inhibitors in Non-tuberous Sclerosis Complex-Related Epilepsy

**DOI:** 10.3389/fneur.2021.639319

**Published:** 2021-02-12

**Authors:** Hannah E. Goldstein, Jason S. Hauptman

**Affiliations:** ^1^Department of Neurological Surgery, University of Washington, Seattle, WA, United States; ^2^Department of Neurosurgery, Seattle Children's Hospital, Seattle, WA, United States

**Keywords:** mTOR, cortical dysplasia, hemimegalencephaly, pediatric epilepsy, non-tuberous sclerosis complex-related epilepsy

## Abstract

Epilepsy affects ~5 out of every 10,000 children per year. Up to one-third of these children have medically refractory epilepsy, with limited to no options for improved seizure control. mTOR, a ubiquitous 289 kDa serine/threonine kinase in the phosphatidylinositol 3-kinase (PI3K)-related kinases (PIKK) family, is dysregulated in a number of human diseases, including tuberous sclerosis complex (TSC) and epilepsy. In cell models of epilepsy and TSC, rapamycin, an mTOR inhibitor, has been shown to decrease seizure frequency and duration, and positively affect cell growth and morphology. Rapamycin has also been shown to prevent or improve epilepsy and prolong survival in animal models of TSC. To date, clinical studies looking at the effects of mTOR inhibitors on the reduction of seizures have mainly focused on patients with TSC. Everolimus (Novartis Pharmaceuticals), a chemically modified rapamycin derivative, has been shown to reduce seizure frequency with reasonable safety and tolerability. Mutations in mTOR or the mTOR pathway have been found in hemimegalencephaly (HME) and focal cortical dysplasias (FCDs), both of which are highly correlated with medically refractory epilepsy. Given the evidence to date, a logical next step is to investigate the role of mTOR inhibitors in the treatment of children with medically refractory non-TSC epilepsy, particularly those children who have also failed resective surgery.

## Introduction

Epilepsy affects ~5 out of every 10,000 children per year (1, 2), with up to one-third of these children having medically refractory epilepsy. When looking only at children with metabolic or structural epilepsy, as opposed to genetic or idiopathic epilepsy, the number of children who continue to have seizures despite multiple antiepileptic drugs (AEDs) jumps to 50% (3–5). A fraction of these patients are referred for surgical evaluation, and if deemed surgical candidates, ~60–80% become seizure free after epilepsy neurosurgery (6–18). However, for children whose seizures persist despite medical therapy and epilepsy surgery, there are limited to no options for improved seizure control.

mTOR, a ubiquitous 289 kDa serine/threonine kinase in the phosphatidylinositol 3-kinase (PI3K)-related kinases (PIKK) family (19) is dysregulated in a number of human diseases, including tuberous sclerosis complex (TSC) and epilepsy. Inhibition of mTOR reduces cell proliferation, angiogenesis, and glucose uptake by cells in both *in vivo* and *in vitro* studies (20–23). Widely used in the treatment of subependymal giant cell astrocytoma (SEGA) in patients with TSC, including children <2 years of age, mTOR inhibitors have been shown to be beneficial for tumor control and also seizure control in this patient population (24–26). However, there are still limited data on the use of mTOR inhibitors for the treatment of non-TSC epilepsy.

## mTOR as an Anti-epileptic Target

In models of pilocarpine-induced seizures, representative of acquired limbic epilepsy, it has been shown that after pilocarpine injection, levels of phosphorylated S6K in the hippocampus and cortex increase at about 30 min and peak at 1 h (27). This rise in phosphorylated S6K can be blocked by pre-treatment with systemic rapamycin, an mTOR inhibitor, at 5 mg/kg/day for 3 days prior to pilocarpine injection, though the pre-treatment does not affect the severity of the acute seizures. In contrast, pilocarpine-treated animals with recurrent spontaneous seizures who are treated with chronic systemic rapamycin (5 mg/kg/day for 3 days, then every other day for 3 weeks) demonstrate a reduction in seizure frequency and duration during treatment; both of which then gradually increase following withdrawal of rapamycin. In a model of pilocarpine-induced status epilepticus, continuous infusion of rapamycin into the dorsal hippocampus prevented mossy fiber sprouting in the molecular and granular layers that then emerged upon withdrawal of treatment (28). Interestingly, when rapamycin was administered after mossy fiber sprouting began (2 months after seizure onset), no effects were seen. This effect on mossy fiber sprouting has been confirmed by others (27).

In another model of temporal lobe epilepsy induced by kainate injection, elevation in phosphorylated S6K in the hippocampus and cortex was noted at 1 h after kainate injection with a peak at 3–6 h and a return to baseline at 24 h (29). An additional phase of rising phosphorylated S6K levels was noted in the hippocampus only, starting at 3 days after injection, peaking by 5 days, and returning to baseline by 5 weeks. Similar to the studies by Huang et al. (27), when rapamycin was administered systemically at a dose of 6 mg/kg/day for 3 days before injection, the biphasic rise in phosphorylated S6K was blocked, however the severity of the acute seizures was not affected. Furthermore, rapamycin pre-treatment reduced kainate seizure-induced hippocampal cell death, kainate-seizure induced dentate granule cell neurogenesis, supragranular mossy fiber sprouting, and chronic recurrent kainate-induced spontaneous epilepsy. When rapamycin treatment was changed from a pre-treatment to a post-treatment paradigm (6 mg/kg/day for 6 days starting 24 h after onset of kainate status epilepticus, then every other day from that point forward), late phase mTOR activation, mossy fiber sprouting, and chronic kainate-induced spontaneous seizures were all reduced. There was no effect on cell death or neurogenesis (27).

In WAG/Rij rats, a genetic model of absence epilepsy, Russo et al. found that early chronic treatment, sub-chronic treatment, or acute treatment with rapamycin all had anti-absence properties. In this model, bacterial lipopolysaccharide (LPS) endotoxin administration causes an increased inflammatory response which results in an increase in absence seizures. However, with co-administration of rapamycin and LPS, this seizure increase was blocked, suggesting an anti-inflammatory pathway (30).

As expected, in models of TSC where Tsc1 is conditionally deleted from most cortical neurons, both rapamycin and RAD-001 (another mTOR inhibitor) increase survival, improve the histological phenotype (cortical organization, soma size and polarity, and myelination), and reduce seizures (31). Additional work has shown that rapamycin completely reverses the elevated endoplasmic reticulum and oxidative stress that can lead to cell death in Tsc2-deficient hippocampal neurons and Tsc1 deficient brain lysates (32). In another model of cortical dysgenesis in which PTEN is conditionally deleted from cortical neurons, rapamycin administration also improved the histological abnormalities (enlarged, disorganized neurons), reduced abnormal EEG activity, and suppressed the frequency and duration of spontaneous seizures (33).

In an animal model of tuberous sclerosis in which Tsc1 is conditionally deleted primarily in glia, rapamycin had significant beneficial effects (34). When rapamycin was given systemically starting at P14 (before the onset of seizures), astrogliosis was prevented, epilepsy did not develop, and animals did not die prematurely. When rapamycin was begun after the onset of epilepsy (at 6 weeks), seizure frequency was decreased, interictal EEG was improved, and survival was prolonged.

## mTOR Inhibitors in TSC Epilepsy

To date, clinical studies looking at the effects of mTOR inhibitors on the reduction of seizures have mainly focused on patients with a known diagnosis of TSC. In an open-label prospective study, 52 pediatric participants with TSC complicated with epilepsy received rapamycin treatment (1 mg/m^2^/d) for at least 24 weeks (35). In participants who received rapamycin treatment for 24, 48, 72, and 96 weeks, reported seizure free rates were 25% (13/52), 19% (6/31), 29% (5/17), and 25% (3/12), respectively. Importantly, though rapamycin therapy did not always result in complete seizure freedom, prior to rapamycin therapy, the average frequency of seizures was 70.27 times/day and the average number of antiepileptic drugs was 1.30. After 24, 48, 72, and 96 weeks' treatment, the average seizure frequency was reduced to 1.94–2.80 times/day and the mean number of concomitant antiepileptic drugs were reduced to 0.83–0.97 (35).

Everolimus (Novartis Pharmaceuticals) is a chemically modified rapamycin derivative that is currently approved for the treatment of pediatric and adult patients with TSC who have surgically inaccessible SEGAs. In 2010, Krueger et al. reported the first large study evaluating everolimus in TSC (36). Expanding upon their findings with the phase 3 EXIST-1 trial, followed by further analyses, Franz et al. reported sustained efficacy in reducing the size of the SEGAs, seizure reduction, and reasonable safety and tolerability, though the study was not sufficiently powered to prove a positive effect on epilepsy (37–41).

The most relevant iteration of the everolimus trials for TSC was EXIST-3, a double-blind placebo-controlled study evaluating everolimus as an adjunctive therapy for treatment-resistant focal-onset seizures in TSC (42). Participants were assigned to placebo, low-dose everolimus (3–7 ng/mL), or high-dose everolimus (9–15 ng/mL). In the study, the median percentage reduction in seizure frequency was 29% in the low dose group and 40% in the high-dose group, with reasonable safety and tolerability results (42).

Similarly, in a single-center, open prospective study of 15 pediatric participants with TSC and epilepsy, Samueli et al. (43) reported that 80% of participants treated with everolimus responded with seizure reduction. In another study by researchers at Cincinnati Children's Hospital, 78% of children treated with everolimus reported ≥50% reduction in seizure frequency at 2 years (44). Additional smaller reports have described reduction in seizure frequency in children with SEGA treated with everolimus (45–47).

## mTOR Inhibitors in Non-TSC Epilepsy

A number of structural developmental brain malformations and tumors have been grouped together and named “TORopathies” due to their shared underlying disruptions in the mTOR pathway (48, 49). These include hemimegalencephaly (HME), polyhydramnios, megalencephaly, and symptomatic epilepsy (PMSE) syndrome, gangliogliomas, dysembryonplastic neuroepithelial tumors (DNETs), and focal cortical dysplasias (FCDs). While each of these is a histopathologic diagnosis, a common clinical feature is intractable epilepsy, suggesting that mTOR plays a role in epileptogenesis.

Hemimegalencephaly and focal cortical dysplasias are both structural abnormalities that are the result of malformations during cortical development. Both are highly correlated with medically refractory epilepsy, and have been associated with mutations in mTOR or the PI3K/AKT/mTOR pathway (50–65). PMSE is known to be caused by mutations in the STRADα gene, an upstream inhibitor of mTORC1, resulting in a decrease in the LKB1/AMPK pathway, and ultimately dysregulated mTOR signaling (66, 67). Gangliogliomas and DNETs have also been shown to exhibit irregular mTOR signaling (48, 50, 68–70). Additionally, variants in genes in the mTOR pathway have been seen in patients with sporadic focal epilepsies (71).

Much of the current evidence for the utility of mTOR inhibitors in the treatment of non-TSC epilepsy is still in the pre-clinical phase. Unlike conventional antiepileptic medications, which tend to modulate neurotransmitter receptors or ion channels, mTOR inhibitors seem to indirectly regulate protein synthesis, which in turn affects ion channels, neuronal signaling, synaptic structure and plasticity (49, 72–76). Animal models of limbic epilepsy (27) and absence seizures (30), both discussed previously, have shown that treatment with rapamycin results in decreased seizure activity.

There are some small clinical trials looking at mTOR inhibitors in non-TSC epilepsy. One open-label prospective study enrolled 5 children with PMSE. These children were treated with daily rapamycin (dose adjusted for a target trough blood concentration between 5 and 15 ng/ml). This study reported 4 out of 5 children were seizure free in the preceding 12-month period, with the fifth child having a single seizure during this time (67). Xu et al. (77) reported >50% reduction in seizures in a single patient with hemimegalencephaly after 1 week of treatment with rapamycin. Two ongoing trials are looking at the role of everolimus in the treatment of patients with FCD and medically refractory epilepsy (78, 79).

## Discussion

There is a strong suggestion in the preclinical literature of an association between mTOR activity and epilepsy/epileptogenesis, particularly in malformations of cortical development. This, coupled with the clinical success of mTOR inhibitors in seizure control in TSC epilepsy and the growing body of evidence supporting the dysregulation of PI3K/Akt/mTOR in a variety of pathologies encountered in pediatric epilepsy, such as focal cortical dysplasia (FCD), suggests that the next step is to investigate the role of mTOR inhibitors in the treatment of children with medically refractory epilepsy, particularly those children who have also failed resective surgery. To this end, our research group is currently studying ABI-009 (nab-rapamycin) in a prospective, phase 1 safety trial to investigate the safety, tolerability, seizure control, and quality of life in participants with medically refractory epilepsy who failed epilepsy surgery (80). Additionally, there are ongoing trials looking at the role of everolimus in the treatment of patients with FCD and medically refractory epilepsy (78, 79).

While there remains more to learn and understand about the role of the mTOR pathway in epilepsy, as well as the underlying cause of seizures in many children with sporadic epilepsy, current data suggest that intervention along this pathway may lead to a reduction in seizure frequency, if not complete seizure freedom. This is of great value for children with medically and surgically refractory epilepsy, for whom even a 50% reduction in seizures can result in considerable improvements in quality of life and overall cognitive development.

## Author Contributions

All authors contributed to the design, writing, critical revision of this review, and approved the final version of the manuscript.

## Conflict of Interest

JH is a consultant for Medtronic. The remaining author declares that the research was conducted in the absence of any commercial or financial relationships that could be construed as a potential conflict of interest.
